# Variability in Hand Surgery Training Among Plastic and Orthopaedic Surgery Residents

**DOI:** 10.5435/JAAOSGlobal-D-21-00138

**Published:** 2022-01-04

**Authors:** Edward J. Testa, Sebastian Orman, Michael A. Bergen, Lauren V. Ready, Neill Y. Li, Joseph A. Gil

**Affiliations:** From the Department of Orthopaedic Surgery, Brown University/Warren Alpert School of Medicine, Providence, RI (Dr. Testa, Dr. Orman, Dr. Bergen, Dr. Li, and Dr. Gil), and Brown University/Warren Alpert School of Medicine, Providence, RI (Dr. Ready).

## Abstract

**Background:**

A career in hand surgery in the United States requires a 1-year fellowship after residency training. Different residency specialty programs may vary in case volume. The purpose of this study was to characterize variation in hand surgery training within and between orthopaedic and plastic surgery residents.

**Methods:**

Publicly available hand surgery case logs for graduating orthopaedic and plastic surgery residents during the 2010 to 2011 to 2018 to 2019 academic years were obtained through the Accreditation Council of Graduate Medical Education. Student *t*-tests were used to compare mean case volumes among several categories between plastic surgery (PRS) and orthopaedic surgery (OS) residents. Intraspecialty variation was assessed by comparing the 90th and 10th percentiles in each category.

**Results:**

A total of 6,254 orthopaedic and 1,070 plastic surgery graduating residents were included. The mean hand surgery case volume for orthopaedic residents (OS 247.0) was significantly lower than that for plastic surgery residents (PRS 412.0) (*P* < 0.0001). Orthopaedic residents performed more trauma cases (OS 133.2, PRS 54.5; *P* < 0.0001) but fewer nerve repairs (OS 3.3, PRS 28.5 *P* < 0.0001) and amputations (OS 6.4, PRS 15.8; *P* < 0.0001). Nerve decompression case volumes were similar between the two specialties (OS 50.2, PRS 47.3; *P* = 0.34). Case volumes among orthopaedic residents varied considerably in amputations and among plastic surgery residents in replantation/revascularization procedures.

**Conclusions:**

Orthopaedic surgery residents performed significantly more trauma cases than plastic surgery residents did, but fewer overall cases, nerve repairs, and amputations, while nerve decompression volumes were similar between specialties. This information may help inform residency and fellowship directors regarding areas of potential training deficiency.

The Accreditation Council for Graduate Medical Education (ACGME) currently mandates hand surgery training for both orthopaedic surgery (OS) and plastic surgery residents (PRS). Although the ACGME specifies case minimums to ensure proficiency, variability in requirements by each specialty exists.^1^ Silvestre et al.^2^ found that orthopaedic surgeons in hand fellowship acquire more cases in fracture management, whereas plastic surgeons in hand fellowship perform more microsurgical cases. While the cited literature focuses on fellowship, hand surgery training during residency may also vary by case volume and variety.

The overarching differences among plastic surgery and orthopaedic surgery residencies are substantial and thus may lead to differences in hand surgery training during residency. In a study evaluating the opinions of orthopaedic and plastic surgery residency program directors regarding essential aspects of hand surgery training, plastic surgery programs valued soft-tissue injury and reconstruction more highly than their orthopaedic counterparts.^3^ Moreover, differences in residency program structure and resident interest inevitably result in varied resident experiences. For example, orthopaedic surgery case volumes, including exposure to hand surgery, are widely variable from resident to resident.^1,4^ Because surgeon case volume is associated with surgical outcomes, evaluating hand surgery training before fellowship is critical.^5,6^

Therefore, we sought to elucidate trends in hand surgery training in orthopaedic and plastic surgery residencies using case logs published by the ACGME. Furthermore, we report the intraspecialty hand surgery case volumes and variability for each respective residency. We hypothesized that orthopaedic residents perform more bony trauma and fracture-related cases compared with microsurgical work related to tissue reconstruction and nerve repair.

## Methods

We evaluated publicly available ACGME case logs for graduating orthopaedic and plastic surgery residents from 2010 to 2019.^7^ Cases logged are a compiled total of all cases performed during residency as reported by the resident. The following data for various hand surgery procedures were manually extracted: case type, total number, 10th/90th case log percentile, mean values, and standard deviations. We also noted the total number of residency programs and individual residents per year for each specialty.

**Figure 1:**
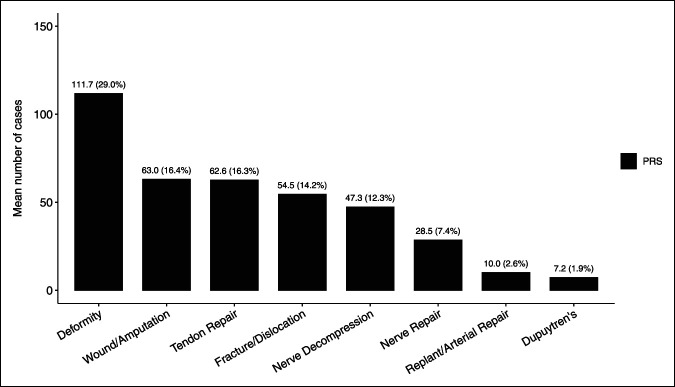
Graphical distribution of hand cases performed during plastic surgery (PRS) residency by number and percentage of total. Total mean number of cases (n) = 412.0.

**Figure 2:**
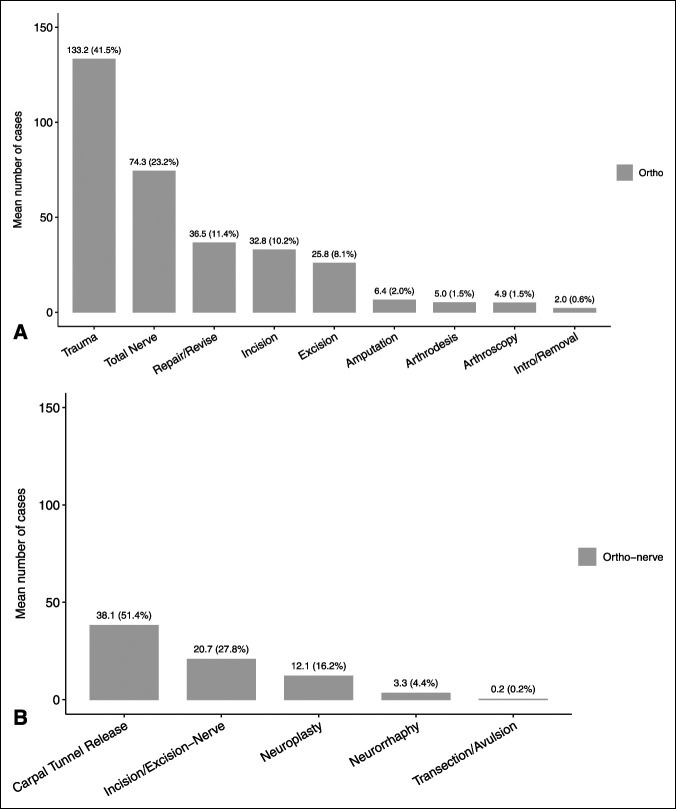
**A**, Graphical distribution of hand cases performed during orthopaedic surgery residency. Total mean number of cases (n) = 247.0. **B**, Further categorization of nerve-related cases performed during orthopaedic surgery (ortho) residency. Total mean number of nerve cases (n) = 74.4. Note that trauma refers to fracture/dislocation cases plus closed reduction/manipulation cases.

The ACGME classifies surgical case logs into several areas, which are then subclassified into procedures.^7^ The areas evaluated for orthopaedic hand training were as follows: hand, forearm/wrist, microvascular, and nervous system. Nervous system procedures such as carpal tunnel release, cubital tunnel release, neuroplasty, and nerve repair were included for analysis. Examples of hand procedures for orthopaedic residents include incision, excision, introduction or removal, repair/revision/reconstruction, manipulation, arthrodesis, and arthroscopy, among others. The areas evaluated by plastic surgery hand training were as follows: hand and upper extremity wounds requiring reconstruction, tendon, nerve injury, fracture or dislocation, Dupuytren contracture, nerve decompression, arterial repair, revascularization, or replantation of digit, hand, or upper extremity, and other deformity or disease process. Figures 1 and 2 present a representation of procedures broken down by category.

The following procedures were logged for direct comparison between specialties: total procedures, nerve repair, nerve decompression, amputation, and trauma. Only these categories were available for direct comparison between the specialties. For total procedures, the cumulative hand and forearm/wrist cases for orthopaedics were compared with total hand cases for plastic surgery. For nerve repair, orthopaedics included cases coded as neurorrhaphy, while plastic surgery included nerve repair. The amputation category was directly comparable between the two specialties. Trauma cases included all hand and wrist/forearm cases for orthopaedics coded as fracture/dislocation and manipulation (ie, closed reduction) and all “fracture or dislocation cases for plastic surgery. Note that trauma refers to bony trauma in orthpaedics, as opposed to tendon, blood vessel, or nerve injuries in plastic surgery. Interspecialty variation (defined as comparisons between orthopaedics and plastic surgery) in case log volume was analyzed using a Student *t*-test or Wilcoxon signed-rank test when appropriate with a cutoff of *P* < 0.05 to represent statistical significance.

To analyze intraspecialty variation (defined as comparisons made within the specialty of either orthopaedics or plastic surgery), the 90th and 10th percentile case volumes for each area were compared between orthopaedic and plastic surgery residents over the academic years of 2011 to 2012 to 2018 to 2019. Fold differences were calculated using the following formula: 90th percentile/10th percentile = fold change.^1^ Linear regression analysis was performed to analyze trends in intraspecialty variation over the study period. A cutoff of a 1,000% change in volume, as previously described by Silvestre et al.,^2^ was used as a threshold to demonstrate substantial variability among the 90th and 10th percentiles. The percentage change was calculated for each category using the following formula: 90th percentile/10th percentile × 100 = percentage change.^2^

We also sought to determine the relationship between case volumes of the 10th, 30th, 70th, and 90th deciles and case log minimum values over the years studied. Both OS and PRS used case minimum requirements in 2014.^8,9^ Of note, OS has only two case minimums within the realm of hand surgery, which were carpal tunnel releases and forearm/wrist closed reduction/manipulation, while PRS use case volume minimums in all categories reported in this study. Absolute and relative volume differences were calculated and averaged among the years studied to determine potential areas of case volume discrepancies among high and low volume residents.

## Results

**Figure 3:**
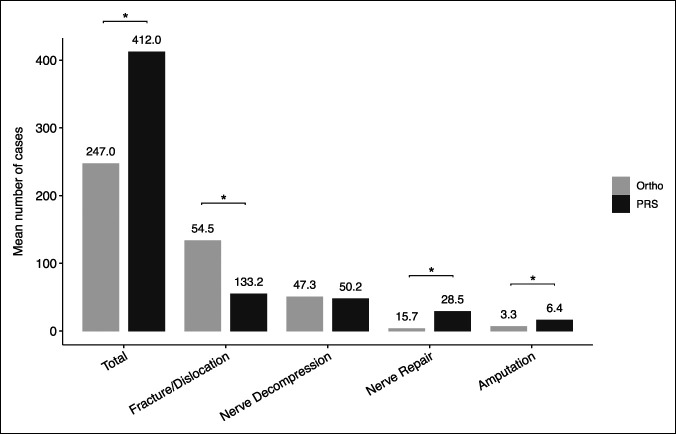
Graphical comparison of relevant categories between orthopaedic and plastic surgery residents. *P < 0.05 for *t*-test comparison between groups. Ortho = orthopaedic surgery, PRS = plastic surgery.

Case logs from a total of 6,254 graduating orthopaedic and 1,070 graduating plastic surgery residents were available for inclusion in this study. Annual trends in graduating residents are described in Table 1. Case averages by category are shown in Figures 1–3.

**Table 1:** Number of Orthopaedic Surgery and Plastic Surgery Residency Programs and Total Residents in the United States During the Academic Years 2011 to 2019

Graduation Year	Orthopaedics		PRS	
	Programs (n)	Residents (n)	Programs (n)	Residents (n)
2011	148	650	69	141
2012	149	675	64	142
2013	150	678	38	75
2014	151	684	65	130
2015	151	699	65	133
2016	153	705	65	133
2017	156	709	64	128
2018	154	729	55	104
2019	154	725	47	84

PRS = plastic surgery

### Interspecialty Analysis

**Figure 4:**
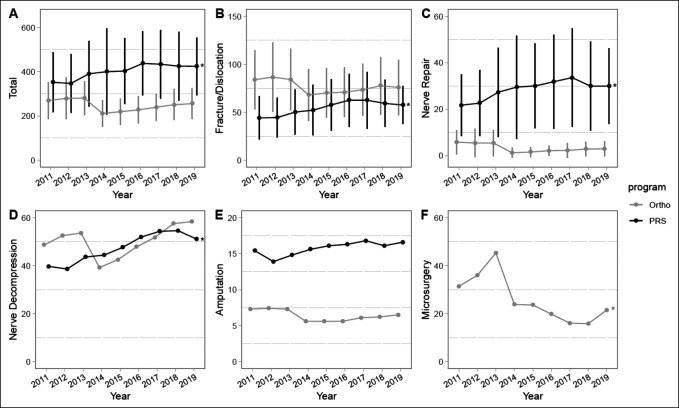
Graphical representation showing mean total case volume (**A**) and mean case volume for four comparable categories (**B**–**E**) between plastic surgery (PRS) and orthopaedic surgery (ortho) residents during the entirety of residency training from academic years 2010 to 2011 to 2018 to 2019. **F**, Mean volume of microsurgery cases completed by orthopaedic residents during their training. Standard deviations marked with vertical bars, when the data were available. Significant differences between specialties (*P* < 0.05) noted in mean case volumes for the following categories over the entire study period: total, amputation, fracture/dislocation, and nerve repair. *P < 0.05 for linear regression trend over time.

Orthopaedic surgery residents graduating between the academic years of 2010 to 2011 and 2018 to 2019 performed significantly fewer total hand cases than plastic surgery residents (OS 247.0, PRS 412.0, *P* < 0.0001) (Figure 4, A). Orthopaedic residents performed more trauma cases (OS 133.2, PRS 54.5; *P* < 0.0001) (Figure 4, B) but fewer nerve repairs (OS 3.3, PRS 28.5 *P* < 0.0001) and amputations (OS 6.4, PRS 15.8; *P* < 0.0001) (Figure 4, E). Regarding the nervous system, orthopaedic surgery residents performed a comparable number of nerve decompressions of that by plastic surgery residents (OS 50.2, PRS 47.3, *P* = 0.34) (Figure 4, D), while plastic surgery residents performed significantly more nerve repairs (OS 15.7, PRS 28.5, *P* < 0.0001) (Figure 4, C). Plastic surgery residency total hand case volume and fracture/dislocation, nerve repair, and nerve decompression case volumes demonstrate a significant trend of increasing case volume over the study period, while these trends were not observed in the orthopaedic surgery cohort (Figure 4).

### Intraspecialty Analysis

Fold differences of the 90th to 10th percentile comparison for each case category for either specialty are summarized in Tables 2 and 3. The hand/finger amputation category for orthopaedic surgery (1,278% change) and replantation/arterial repair/revascularization category for plastic surgery (1,119% change) demonstrate substantial variability between the 90th and 10th percentiles of residents (Table 2). For orthopaedic surgery, several categories contained zero cases for the 10th percentile groups and thus were not able to be analyzed because the used equation resulted in a denominator of zero. These categories included introduction or removal (hand and forearm/wrist), trauma manipulation (hand), arthrodesis (hand and forearm/wrist), other (hand, forearm/wrist, and nervous system), amputation (forearm/wrist), arthroscopy, transection/avulsion (nervous system), neurorrhaphy, incision/excision (nervous system), and microsurgery.

**Table 2:** Fold Difference Between 10th and 90th Percentile of Cases for Various Categories in Orthopaedic Hand Surgery Training

Procedure	2011	2012	2013	2014	2015	2016	2017	2018	2019	*P*	Average Percentage Difference
Hand/finger											
Incision	4.5	4.5	4.3	4.2	5.3	4.3	4.4	4.2	4.0	0.37	442
Excision	5.8	5.8	5.8	6.0	5.8	6.0	6.5	6.3	5.8	0.21	597
Repair/revise/reconstruct	5.1	4.8	4.6	5.2	4.3	4.5	4.7	5.3	5.2	0.65	486
Fracture/dislocation	4.9	5.1	5.8	4.3	5.1	5.1	4.9	4.9	4.8	0.55	499
Amputation	15.0	15.0	15.0	11.0	11.0	11.0	12.0	12.0	13.0	0.10	1,278
Total	3.3	3.1	3.1	2.8	3.1	3.2	2.8	3.1	2.9	0.20	304
Forearm/wrist											
Incision	7.0	7.0	7.0	10.0	10.0	10.0	12.0	11.0	12.0	<0.01^a^	956
Excision	5.8	4.8	4.8	5.0	6.3	4.8	5.0	5.0	5.0	0.59	517
Repair/revise/reconstruct	7.0	6.5	6.7	6.7	6.7	7.3	5.8	6.5	6.5	0.35	663
Fracture/dislocation	3.2	3.2	3.2	3.0	3.0	3.1	3.1	3.1	3.0	0.07	310
Manipulation	37.5	19.3	4.2	4.0	3.6	3.5	3.7	3.7	3.6	0.03^a^	923
Total	2.7	2.6	2.3	2.4	2.2	2.3	2.2	2.3	2.2	<0.01^a^	236
Nervous system											
Neuroplasty	7.0	6.0	6.0	10.5	8.0	8.5	9.5	7.3	7.3	0.43	779
Carpal tunnel	7.2	5.1	4.9	5.5	4.6	4.3	4.5	4.0	4.1	<0.01^a^	491
Total	5.1	3.9	3.5	4.4	4.1	3.8	3.8	3.6	3.3	0.04^a^	395

a *P* < 0.05 denotes significant change over time in each category, as measured by linear regression. N/A = Not applicable.

**Table 3:** Fold Difference Between 10th and 90th Percentile of Cases for Various Categories in Plastic (PRS) Hand Surgery Training

Procedure	2011	2012	2013	2014	2015	2016	2017	2018	2019	*P*	Average Percentage Difference
PRS											
Amputation	6.0	5.2	5.2	8.0	4.4	4.3	3.1	3.2	3.6	0.04^a^	478
Wound reconstruction	3.7	3.6	4.7	4.5	3.0	3.1	3.1	2.8	2.9	0.05	349
Tendon	3.6	4.2	3.4	4.3	3.2	2.9	3.6	3.4	2.9	0.11	350
Nerve injury	5.0	4.7	6.0	5.5	4.4	4.2	3.9	4.6	3.9	0.05	469
Fracture/dislocation	3.5	3.1	3.1	3.5	2.9	2.9	3.2	2.6	2.3	0.01^a^	301
Dupuytren disease	7.5	6.5	8.5	7.0	7.5	7.0	5.3	6.0	8.0	0.48	703
Nerve decompression	4.8	5.3	4.1	4.4	4.1	4.6	5.7	4.7	3.9	0.69	462
Replant/arterial repair/revascularize	19.0	8.5	24.0	20.0	6.3	7.3	5.8	4.8	5.0	0.03^a^	1,119
Arthroplasty/arthrodesis	10.5	11.0	8.3	11.0	5.5	6.0	6.8	5.0	5.5	<0.01^a^	773
Congenital	N/A	N/A	N/A	N/A	13.0	6.5	7.5	6.5	7.5	N/A	820
Neoplasm	4.3	5.6	6.7	5.1	4.2	3.8	4.4	3.7	3.0	0.04^a^	453
Total	2.4	N/A	N/A	N/A	2.6	2.3	2.6	2.2	2.1	N/A	237

a *P* < 0.05 denotes significant change over time in each category, as measured by linear regression.

Using the top and bottom 10th percentiles, orthopaedic surgery residents demonstrated a significant decrease in case volume variability between the academic years of 2011 to 2012 and 2018 to 2019 in following case categories: manipulation of forearm/wrist (*P* = 0.03), total wrist/forearm cases (*P* = 0.009), carpal tunnel releases (*P* = 0.006), microsurgery (*P* = 0.02), and total nerve cases (*P* = 0.044) (Table 2). Incision of the forearm/wrist was the sole category demonstrating increasing variability (*P* = 0.0003) (Table 2). For plastic surgery residents, decreasing variability was noted in amputation (*P* = 0.04), fracture/dislocation (*P* = 0.01), replantation/arterial repair/revascularization (*P* = 0.03), arthroplasty (*P* = 0.005), and neoplasm (*P* = 0.04) case categories (Table 3).

### Case Volume Relative to Minimum Accreditation Council of Graduate Medical Education Required Cases

For orthopaedics, the 10th percentile group demonstrated fewer than the minimum required cases by ACGME standard in closed reduction of the wrist/forearm. For plastic surgery, the 10th percentile group demonstrated fewer than the minimum within the categories of amputation, fracture/dislocation, replantation, and congenital cases (Table 4).

**Table 4:** Mean Annual Hand Surgery Case Volume for Orthopaedic and Plastic Surgery Residents Compared With ACGME Minimum

Procedure	Case Minimum	Percentile			
		10th	30th	70th	90th
OS					
Carpal tunnel	10	3.7 (37%)	13.5 (135%)	39.4 (394%)	69.7 (697%)
Closed reduction, forearm/wrist	20	**−0.3 (−3%)**	9.3 (93%)	36.0 (360%)	67.0 (670%)
PRS					
Amputation	7	**−0.6 (−8%)**	2.9 (41%)	11.4 (163%)	21.7 (310%)
Wound reconstruction	23	7.7 (33%)	21.2 (92%)	49.8 (216%)	81.4 (354%)
Tendon	22	7.9 (36%)	19.9 (90%)	50.9 (231%)	81.1 (369%)
Nerve injury	10	1.2 (12%)	7.1 (71%)	23.3 (233%)	41.3 (413%)
Fracture/dislocation	30	**−0.8 (−3%)**	8.4 (28%)	32.9 (110%)	56.8 (189%)
Dupuytren disease	2	0.2 (11%)	1.6 (78%)	6.3 (317%)	13.3 (667%)
Nerve decompression	16	2.0 (13%)	13.0 (81%)	38.4 (240%)	66.1 (413%)
Replantation	4	**−1.3 (−33%)**	0.7 (17%)	7.6 (189%)	17.1 (428%)
Arthroplasty	3	0.2 (7%)	3.0 (100%)	10.2 (341%)	19.8 (659%)
Congenital	2	**−1.0 (−50%)**	0.4 (22%)	4.7 (233%)	10.8 (539%)
Neoplasm	8	0.1 (1%)	4.9 (61%)	15.8 (197%)	27.1 (339%)
Total hand cases	122	144.8 (119%)	223.0 (183%)	368.0 (302%)	484.0 (397%)

ACGME = Accreditation Council for Graduate Medical Education, OS = orthopaedic surgery; PRS = plastic surgery residents

Values for each decile are represented as absolute difference followed by relative difference (in parentheses). Bold values represent average case volume lower than the case minimum.

## Discussion

Orthopaedic surgery residents performed more hand, wrist, and forearm bony trauma cases compared with their plastic surgery colleagues. On the contrary, plastic surgery residents performed 60% more total hand procedures, with significantly more nerve repairs and amputations completed. Both specialties performed a similar number of nerve decompression cases. Several areas of intraspecialty variability were observed among plastic and orthopaedic surgery residents, with a trend toward decreasing case volume variability in several case categories.

Peripheral nerve repair is a technically demanding procedure, which is commonly performed by hand surgeons. Plastic surgery residents performed an average of more than 28 nerve repairs during residency, compared with only 3.3 for orthopaedic residents (*P* < 0.0001) (Figure 3). Plastic surgery residents must perform at least 10 nerve repairs to graduate residency, whereas no minimum number of nerve repairs is required for orthopaedic residents.^8,9^ During hand surgery fellowship, plastic surgery fellows perform more peripheral nerve surgeries than orthopaedic fellows, further widening the training gap.^2^ The relevance of these findings is underscored by the understanding that increased case volumes are indicative of resident competence and comfort in the operating room.^10^ Given this information, orthopaedic hand fellowships and residency programs should similarly place additional emphasis on nerve repair surgery to ensure orthopaedic hand fellows are prepared for independent practice.

Although microsurgery is performed relatively infrequently during orthopaedic residency training, it is a vital surgical skill to the adept hand surgeon, and high volume training is essential to obtain microsurgical competence.^11^ Training in microsurgery among orthopaedic residents varies substantially, with the top 10th percentile of residents logging more than 6 times more cases than the bottom 50th percentile.^12^ Literature has also noted that between 2007 and 2013, graduating orthopaedic residents increased in microsurgical case volume.^12^ However, in recent years, orthopaedic residents have been performing significantly fewer microsurgical cases, while case volume differences among high and low volume residents have significantly decreased (Figure 4, F). Although surgical case volume is positively correlated with improved outcomes,^6^ the importance of such volume during residency training has not been fully elucidated and would be an interesting area of future research.

The hand is the most commonly injured body part, with an estimated 1 in 88 chance of a resident of the United States presenting to the emergency department with an upper extremity injury in any given year.^13,14^ Despite performing fewer overall hand cases, orthopaedic residents record significantly more fracture and dislocation cases than plastic surgery residents. During hand surgery fellowship, orthopaedic-trained fellows also perform more open hand, wrist, and forearm cases, while plastic surgery fellows performed more closed treatments of fractures or dislocations, further separating the training experience between these two specialties.^2^ Orthopaedic residency affords trainees not only hand and upper extremity surgery rotations but also dedicated trauma rotations during which residents perform fixation of forearm, wrist, and hand fractures. By contrast, plastic surgery residents have only two main areas of bony trauma experience—traumatic hand and upper extremity, and facial trauma, limiting their experience to hand surgery rotations, facial fractures, and on-call cases.

Such training patterns may also relate to trends in clinical practice because orthopaedic surgeons repair nearly 10 times as many fractures and dislocations of the carpus and forearm than their plastic surgery colleagues.^15^ Only approximately half of plastic surgery residents decide to pursue fellowship training, whereas at least 90% of orthopaedic residents enter fellowship.^16,17^ Our data show that residents completing plastic surgery programs complete on average more than 400 hand cases and 28 nerve repairs compared with orthopaedic residents who complete approximately 240 hand cases and 3 nerve repairs. This discrepancy in volume may explain why plastic surgery residents tend to enter practice directly after residency rather than undertaking a dedicated year of hand surgery training, as is more often the case with the orthopaedic resident.

Case volume variability among residents within the same specialty may elucidate areas of training deficiencies at low volume programs. When comparing the top and bottom 10th percentile of residents, orthopaedic residents varied substantially in hand and finger amputations while plastic surgery residents varied in replantation/revascularization procedures (Tables 2 and 3). For orthopaedics residents, 10 case categories were noted in which the 10th percentile of programs averaged zero cases, while this was only noted in one category for plastic surgery in the 10th percentile. This precluded quantitatively analyzing such categories for variability by our methods. However, these categories averaging zero cases in lower volume programs may provide a strong indicator of substantial case variability, despite being unable to quantify as such by our methods. Thus, deficiencies may exist in the complete breadth of hand surgery training at some residency programs, which has been cited as a recent concern.^4^

Evaluating variability over time, several categories were observed for both residency types with notable decreases in case volume variability. This pattern was noted for manipulation (ie, closed reduction) of the wrist/forearm, incision (forearm/wrist), total wrist/forearm cases, carpal tunnel releases, and total nerve cases in orthopaedics, and amputation, fracture/dislocation, replantation/arterial repair/revascularization, arthroplasty/arthrodesis, and neoplasm for plastic surgery among the top and bottom 10th percentiles. In accordance with literature, these results indicated a trend toward decreasing hand surgery case volume variability in residents, reproducing a reassuring finding for hand surgery training in residency.^1^

The ACGME used case volume minimums for both orthopaedic surgery and plastic surgery residents in 2014,^8,9^ thus establishing surgical thresholds in particular case categories necessary to complete residency training.^8,9^ Our results demonstrated that several categories of either orthopaedic or plastic surgery training within the lowest decile of resident case volume did not reach the annual minimum volume threshold on average over the years studied (Table 4). For orthopaedics, this category was closed reduction of the wrist/forearm, while for plastic surgery, these categories included amputation, fracture/dislocation, replantation, and congenital cases. On the contrary, the highest decile residents in orthopaedics performed more than 600% times the minimum case volumes, while this group of plastic surgery residents ranged from 189% to 667% greater than the case minimum over all categories (Table 4). However, when considering the 30th and 70th percentile of residents, these groups reached case volume minimum thresholds in all categories. These findings highlight the case categories that may remain deficient in case volume for residents at the lowest volume of training and may provide areas of improvement for residency program directors. Only two hand surgery–related case categories among orthopaedic residents had case volume minimums (carpal tunnel release and closed reduction of wrist/forearm), limiting our analysis to these two categories, while plastic surgery had minimums for all categories.

Our study contains several limitations. Importantly, directly comparing plastic surgery to orthopaedic surgery residents was not appropriate for several case categories. The ACGME categories for orthopaedics are less specific than those for plastic surgery, and no standardized grouping or reporting criteria are present to ensure appropriate comparisons among different residency types. Regardless, the categories compared by our study remain of educational importance. Nerve decompressions and trauma cases are among the most commonly performed hand procedures by fellowship-trained hand surgeons,^18^ making this comparison useful to residency directors when optimizing a curriculum for residents interested in pursuing hand surgery fellowship. Moreover, the ACGME does not publicly release data for all residency programs. Over the years studied, an average of approximately 163 unique orthopaedic residency programs graduating approximately 699 graduates annually and approximately 90 plastic surgery programs graduating 210 plastic surgery residents annually were found.^19–21^ As a result, our data represent approximately 98% of orthopaedic residents and 95% of orthopaedic surgery residencies, while only capturing 55% of plastic surgery residents and 65% of plastic surgery residencies (Table 1). Thus, our limited data of plastic surgery residents may result in sampling bias, potentially limiting the validity of our results. Finally, this analysis depended on the reliability and consistency of residents in logging their cases. Therefore, our results may be affected by residents who fail to properly code or log their cases.

## Conclusion

Using ACGME resident case logs, notable intraspecialty and interspecialty variations in hand surgery training patterns among graduating orthopaedic and plastic surgery residents were observed. Orthopaedic residents perform more bony trauma surgeries, while plastic surgery residents perform more nerve repairs and amputations. Future research into competency-based case volume minimum requirements may help educators minimize variation in hand surgery experience and optimize resident preparation for fellowship and subsequent clinical practice.
